# Journal of clinical monitoring and computing 2016 end of year summary: monitoring cerebral oxygenation and autoregulation

**DOI:** 10.1007/s10877-017-9980-7

**Published:** 2017-01-24

**Authors:** Thomas W. L. Scheeren, Bernd Saugel

**Affiliations:** 1 Department of Anesthesiology, University Medical Center Groningen, University of Groningen, Groningen, The Netherlands; 2 Department of Anesthesiology, Centre of Anesthesiology and Intensive Care Medicine, University Medical Centre Hamburg-Eppendorf, Martinistrasse 52, 20246 Hamburg, Germany

**Keywords:** Monitoring, Tissue oxygenation, Cerebral blood flow, Autoregulation, Near infrared spectroscopy, Cerebral oximetry

## Abstract

In the perioperative and critical care setting, monitoring of cerebral oxygenation (ScO_2_) and cerebral autoregulation enjoy increasing popularity in recent years, particularly in patients undergoing cardiac surgery. Monitoring ScO_2_ is based on near infrared spectroscopy, and attempts to early detect cerebral hypoperfusion and thereby prevent cerebral dysfunction and postoperative neurologic complications. Autoregulation of cerebral blood flow provides a steady flow of blood towards the brain despite variations in mean arterial blood pressure (MAP) and cerebral perfusion pressure, and is effective in a MAP range between approximately 50–150 mmHg. This range of intact autoregulation may, however, vary considerably between individuals, and shifts to higher thresholds have been observed in elderly and hypertensive patients. As a consequence, intraoperative hypotension will be poorly tolerated, and might cause ischemic events and postoperative neurological complications. This article summarizes research investigating technologies for the assessment of ScO_2_ and cerebral autoregulation published in the Journal of Clinical Monitoring and Computing in 2016.

## Introduction

In the perioperative setting, particularly in patients undergoing cardiac surgery, monitoring of cerebral oxygenation (ScO_2_) enjoys increasing popularity in recent years. The rationale behind its use is the attempt to early detect cerebral hypoperfusion, which may be caused by systemic hypotension or the use of the cardiopulmonary bypass, and thereby prevent cerebral dysfunction and postoperative neurologic complications [1]. In addition to the widespread use in cardiac anaesthesia and postoperative care, ScO_2_ monitoring has spread over the whole range of perioperative and critical care settings, [1] examples of which are given below.

Autoregulation of blood flow is a key feature of the human cerebral vascular system to assure adequate oxygenation and metabolism of the brain under changing physiological conditions. This is essential since due to its high metabolic activity, the brain does not tolerate hypoxia or hypoperfusion. The autoregulation of cerebral blood flow (CBF) provides a steady flow of blood towards the brain by altering vascular resistance through complex myogenic, neurogenic, and metabolic mechanisms. This autoregulatory control mechanism therefore buffers any variations in mean arterial blood pressure (MAP) and cerebral perfusion pressure (CPP) and is effective in a MAP range between approximately 50–150 mmHg, defining the lower (LLA) and upper limit of autoregulation (ULA), respectively. This range of intact autoregulation may, however, vary considerably between individuals, and shifts to higher thresholds have been observed in elderly and hypertensive patients. At the blood pressure extremes, i.e. below the LLA and above the ULA, the cerebral vasculature is no longer able to adapt its resistance in response to further blood pressure changes. The clinical consequence is for instance that intraoperative hypotension (with MAP values below the LLA) will be poorly tolerated, and might cause ischemic events and postoperative neurological complications. Therefore, besides ScO_2_, the patient`s autoregulatory status might be an important monitoring issue, which could give the clinician important prognostic information on neurologic outcome and allow for adequate therapeutic measures to be taken.

In this regard, the Journal of Clinical Monitoring and Computing (JCMC) welcomes research investigating technologies for the assessment of ScO_2_ and cerebral autoregulation (CA). In this review, we summarize and discuss papers about monitoring of ScO_2_ using near infrared spectroscopy (NIRS) as well as publications on CA printed last year in the JCMC.

## Near infrared spectroscopy

The NIRS technology was investigated in several studies published in the journal in 2016. The first two papers comprise volunteer studies analysing the NIRS signal in depth. In the August issue, Colquhoun et al. [2] performed a frequency domain analysis of NIRS signals recorded in 20 volunteers in order to separate the arterial and venous contribution to the signal. The background of their study is the fact that most current commercially available oximetry devices do not discriminate between arterial and venous blood in the investigated sample volume, and assume a fixed ratio of arterial to venous blood varying from a 70:30 ratio to a 80:20 ratio, depending on the device used [3]. Yet, this assumption, which is mainly based on anatomical evidence, may not always be true, and should be more weighted towards arterial haemoglobin saturation, as recently shown [4]. The authors hypothesized that frequency domain analysis of photoplethysmographic (PPG) and NIRS signals may discriminate between arterial and venous blood. In order to alter the contribution of the venous part of the signal, the authors used an impedance threshold device (ITD) in their volunteers, which amplifies the effect of respiratory pressures on blood flow by increasing intrathoracic pressure and thereby might temporarily alter the arterial to venous blood ratio within the brain. ScO_2_ was measured via a special two-wavelengths portable NIRS device, which is based on spatially resolved spectroscopy techniques. After baseline measurements, the ITD was applied and a second set of measurements was taken. For analysis, the spatially resolved absorbance waveforms were transformed into the frequency domain and relative concentrations of oxygenated and deoxygenated haemoglobin were calculated by using the two wavelengths in seven frequency domains for each individual. While the ITD increased ScO_2_ by 3.6% on average, the induced low and high frequency modulations in the NIRS signals could not be exclusively attributed to arterial and venous blood, respectively. Obviously, the low and high frequency components of both the PPG and NIRS waveforms contain contributions from both arterial and venous blood, the relative amounts of which are not known. Of note, since the NIRS waveforms show the same respiratory variations as the arterial pressure [5] or PPG waveforms [6, 7], they might be used to non-invasively determine fluid responsiveness as well, particularly when peripheral perfusion is compromised.

In the April issue, Hirasawa et al. [8] developed an algorithm that eliminates the influence of skin blood flow in the NIRS signal. Against the background of recent literature showing that scalp and skull blood flow (SSBF) may contaminate the NIRS signal traveling through these structures and thus affect ScO_2_ readings, [9, 10] the authors used a headband cuff, which was placed above the superficial temporal artery and inflated repeatedly to 80 mmHg in 12 healthy volunteers in order to suppress SSBF, as verified by laser Doppler flowmetry. To eliminate SSBF influence on the NIRS-derived cerebral oxygenation, most commercial NIRS devices employ two source-detector distances (mostly between 15 and 30 mm for the short and 40–50 mm for the long distance) and subtract the signal from the short distance-detector (reflecting superficial tissues) from that of a long-distance (reflecting brain tissue), a method known as spatially resolved NIRS. However, recent literature suggests that this technique does not fully eliminate SSBF influence on the NIRS signal, as shown previously by the group of authors for instance after vasoconstrictor application [10, 11]. Hence, the authors developed an algorithm with an individual correction factor for extracranial blood flow to isolate cerebral oxygenation from the NIRS signal based on suppressed SSBF. This algorithm was then validated against resting conditions during cerebral activation induced by handgrip exercise and a cognitive task. Both interventions did significantly increase SSBF and ScO_2_. Inflation of the headband reduced both SSBF and the original ScO_2_ under all conditions studied, whereas it did not affect the algorithm-estimated ScO_2_. The authors conclude that their algorithm with an individual correction factor successfully eliminated the influence of SSBF on the NIRS signal, allowing for measurement of valid (changes in) cerebral oxygenation. The complexity of their approach will however limit its use to special applications such as physiological studies on cerebral activation.

Five more articles are dealing with the impact of ScO_2_ monitoring on patient management in different clinical settings.

In the April issue, Sorensen et al. [12] report a retrospective study on a ventilation strategy during open abdominal aortic aneurysm repair; in this study, the authors evaluated ScO_2_ and its relation to end-tidal carbon dioxide tension (etCO_2_) during the surgery. This study setting is especially interesting because marked hemodynamic changes very rapidly occur during this surgical procedure (clamping and de-clamping of the aorta). This is challenging with regard to hemodynamic and respiratory support as these clamping/de-clamping manoeuvres also induce changes in the patients’ metabolism (with reduced cardiac output and metabolism during clamping and an increase in partial pressure of carbon dioxide after reperfusion). The authors analysed 44 patients in whom mechanical ventilation was adjusted according to etCO_2_ and ScO_2_ was monitored with NIRS. They report that etCO_2_ and ScO_2_ were kept constant after aortic clamping by reducing minute ventilation (median −0.8 L min). After de-clamping of the aorta, an increase in minute ventilation by a median of 1.8 L min resulted in an increase in ScO_2_ of 2%, while despite the increase in minute ventilation median etCO_2_ increased by 0.5 kPa. From these observations, the authors conclude that ScO_2_ can be kept within reasonable limits by reducing ventilation by about 1 L/min during clamping of the aorta and increasing ventilation by about 2 L/min during reperfusion. This rule of thumb adjustment of ventilator management can be further fine-tuned by ScO_2_ monitoring.

Erdem et al. [13] performed a study (published in the October issue) on the effect of controlled hypotension during elective rhinoplasty on ScO_2_ assessed using NIRS. The authors included 50 adults in whom controlled hypotension was achieved by using total intravenous anaesthesia and nitroglycerin infusion (if needed). The authors defined “cerebral desaturation” as a decrease in ScO_2_ of lower than 80% of individual baseline ScO_2_ for more than 15 s and report that this endpoint occurred in 5 out of the 50 patients. Interestingly, none of the episodes of cerebral desaturation was accompanied with a decrease in the peripheral oxygen saturation or the etCO_2_. Therefore, this interesting study demonstrates that NIRS can indicate marked decreases in ScO_2_ in patients undergoing controlled hypotension even if the peripheral oxygen saturation remains in a normal range.

The relation between hypotension and ScO_2_ was investigated by Sun et al. (see August issue) [14]. In 45 parturients undergoing combined spinal-epidural (CSE) anaesthesia for Caesarean section, the authors studied if hypotensive episodes (defined as a decrease in systolic blood pressure below 80% of baseline) could be predicted by a decrease in ScO_2_. This would be important since hypotension in this setting is frequent (occurring in about 70% of their patients) and may jeopardize both fetus (hypoxia, acidosis) and parturient (nausea, vomiting, syncope), and common prophylactic measures such as volume loading or vasopressor administration failed to significantly reduce its incidence [15]. The authors prospectively observed ScO_2_ (the readings of which were blinded for the anaesthetist in charge) and blood pressure (discontinuously every minute) in 45 parturients not receiving any premedication or prophylactic measures to prevent hypotension. A decrease in ScO_2_ ≥ 5% from individual baseline values was chosen as threshold for prediction of hypotension. ScO_2_ decreased significantly more after CSE anaesthesia in parturients developing hypotension as compared to those without hypotension, probably due to a hypotension-induced reduction in CBF. More important, the decrease in ScO_2_ occurred earlier (about 40 s) than did hypotension, a time span sufficient to take corrective therapeutic measures. But how can ScO_2_ decrease earlier than blood pressure if the decrease in ScO_2_ is caused by the hypotension? The authors try to explain this by reflex upper-body vasoconstriction and reduction in venous return secondary to CSE anaesthesia, but it could also be due to the higher temporal resolution (seconds) of the ScO_2_ signals compared to intermittent blood pressure measurements (minutes). Nevertheless, ROC analysis revealed a decrease in ScO_2_ as a good predictor of hypotension with an optimal threshold value of 4.5% and a positive predictive value of 0.92. If NIRS monitoring should be used for prediction or early detection of hypotension (as suggested by the authors) in a broader scale depends on the costs (of disposable sensors) associated with this kind of monitoring. It might also be argued that intensifying blood pressure monitoring towards continuous measurements (such as currently available with several non-invasive methods) [16] will also enable to prevent or reduce the incidence of hypotension significantly as well.

In the October issue, Kerz et al. [17] report an interesting study investigating the correlation of ScO_2_ measured by continuous-wave NIRS measurements with invasive brain tissue oxygenation measurements (PtiO_2_) in 11 neurosurgical ICU patients. This study approach is interesting because validation data for NIRS—although widely clinically used e.g. in cardiothoracic anaesthesia—are scarce. Interestingly, the authors found very low correlation coefficients for the correlation of NIRS and PtiO_2_; in addition, the predictive capabilities of NIRS for an PtiO_2_ of <15 mmHg were bad (area under the curve of the receiver operating characteristics curve about 0.56). The authors conclude that continuous-wave NIRS does not well correlate with invasively assessed PtiO_2_ values and that NIRS cannot detect episodes of cerebral ischemia. It has to be emphasized, however, that the authors used the continuous-wave NIRS method that is based on intensity alterations of emitted light. Therefore, results cannot unconditionally be transferred to other more sophisticated NIRS methods (such as frequency-domain or time-domain-based measurements). Furthermore, it has to be stressed that NIRS measures the haemoglobin oxygen saturation of the blood within the arterioles and venules with a signal weighting of approximately 20 versus 80% or 25 versus 75%, respectively, depending on the device [3].

Also in the October issue, a case-series by Brodt et al. [18] was published evaluating changes in cerebral oxygen saturation in 10 patients during transcatheter aortic valve replacement under general anaesthesia. As transcatheter aortic valve replacement is used in cardiovascular high-risk patients and includes rapid-frequency ventricular pacing during valve deployment, patients undergoing this procedure are at risk for decreases in ScO_2_. The authors report relatively low baseline ScO_2_ values of 56 ± 7% in their high-risk patients. After induction of general anaesthesia, the authors expectedly observed an increase in ScO_2_. During valve deployment, the mean ScO_2_ was 49 ± 13%. In two patients ScO_2_ decreased more than 20% compared to baseline values. After valve deployment, ScO_2_ returned to baseline values in all of the ten patients (this return to baseline, however, took up to 20 min in three patients (mean 13 ± 10 min)). Unfortunately, this case-series does not give details of the functional neurological status of the patients before and after the intervention. Nevertheless, it illustrates that the sudden decrease in cardiac output by rapid-pacing results in a marked transient decrease in ScO_2_. Strategies to optimize ScO_2_ prior to valve deployment might improve patient safety during transcatheter aortic valve replacement and should be evaluated in future studies.

In the December issue, an interesting systematic review on the use of NIRS during cardiological procedures by Moerman et al. [19] has been published. The authors hypothesized that NIRS monitoring might help improving patient safety in this group of patients with marked risk for cardiovascular complications. Applying a systematic search strategy to search electronic bibliographic databases the authors identified 11 observational studies (no randomized trial was available) and five case reports on the use of NIRS in patients during cardiological procedures (six studies during electrophysiology for arrhythmias, four studies during pediatric catheterization procedures, one study during transcatheter aortic valve implantations); based on these studies the authors assessed the evidence for the use of NIRS. Based on this limited number of available studies (all of which had a low statistical power) the authors conclude that NIRS provides a very quick representation of ScO_2_ and that it might identify changes that could not be predicted from standard hemodynamic monitoring during cardiological procedures. Nevertheless, the authors emphasize that the evidence for improved patient outcome is currently not high enough to generally recommend the use of NIRS for all cardiological procedures.

## Autoregulation of cerebral blood flow

In the June issue of the journal, Goettel et al. [20] addressed this issue and investigated the effect of sevoflurane anaesthesia on CA in 133 patients of two different age groups, a younger (age 18–40 years, n = 49) and an elderly cohort (age ≥ 65 years, n = 84). It is known that volatile anaesthetics impair the CA response in a dose-dependent manner. Therefore, the authors hypothesized that CBF autoregulation would be less effective in older patients as compared to younger study subjects under sevoflurane anaesthesia and expected a shorter autoregulatory plateau due to an increased LLA in older patients. CBF was measured bilaterally by transcranial Doppler (TCD) and blood pressure non-invasively by the finger volume clamp method. Both values were correlated and the linear correlation coefficient Mx taken as a measure of CBF autoregulation, with a Mx of 0 indication intact autoregulation and a positive Mx (approaching 1) indicating loss of autoregulation and pressure-driven CBF. In their prospective observational study, they found a LLA of 66 ± 12 mmHg and 73 ± 14 mmHg in young and older patients, respectively, but no difference in the ULA (70 ± 14 mmHg in older vs. 73 ± 19 mmHg in younger patients, respectively). Hence, the autoregulatory range was substantially smaller than the expected 100 mmHg, and tended to be greater for younger than for older patients (14 ± 10 mmHg vs. 10 ± 9 mmHg). Furthermore, Mx was significantly higher in older compared to younger patients, indicating that CBF autoregulation was less effective in the elderly. The authors conclude that the autoregulatory plateau is shortened substantially in both young and older patients under sevoflurane anaesthesia with approximately 1 MAC as compared to awake subjects, probably due to its vasodilator effects. However, other factors on CA such as patient comorbidity, carbon dioxide levels, cerebral metabolism, and vasoactive agents cannot be excluded. Remarkably, the LLA and ULA, as well as the autoregulatory range were not influenced by the age of anaesthetized patients. The results imply that patients under general anaesthesia are less protected by CA and may be more susceptible to cerebral ischemia or edema.

In an accompanying editorial, Moerman and Absalom [21] point out some weaknesses of the abovementioned study, including the fact that in the majority of their patients (89%), the LLA and/or ULA were not reached, mainly because major fluctuations in blood pressure were prevented. Nevertheless, they acknowledge the importance of the study findings that sevoflurane may alter the position and shape of the CA curve and the implications thereof for an individualized perioperative hemodynamic management.

CA is certainly important in traumatic brain injury (TBI), which may be associated with intracranial hypertension. In the December issue, Kim et al. [22] report the results of their automatic data monitoring for CA. They developed an integrated platform for acquiring and evaluating data necessary for developing predictive models and collected pressure data from 29 TBI patients admitted to their ICU. Subsequently, they used the established pressure reactivity index (PRx), which is based on the assumption that intracranial pressure (ICP) should not directly correlate with arterial blood pressure, and found that it can predict intracranial hypertensive events (defined as ICP increases above 25 mmHg for >5 min) in the hour preceding the event. The accuracy of the prediction based on a certain PRx threshold (i.e. >0.8) was, however, rather low. Furthermore, it has to be shown in future studies if these methods based on retrospective analyses of intracranial hypertensive events that had already occurred can be transferred to predict and probably prevent such events.

In the October issue, Montgomery et al. [23] performed a secondary analysis on a porcine dataset (containing NIRS and systemic blood pressure data) to investigate data clustering methods as a technique for determining the LLA. This way they question the traditional approach of using binned data to assess CA functionality. A non-invasive method using NIRS technology instead of TCD was used as reference. For this, the ScO_2_ and MAP values were correlated, and, similar to the above mentioned Mx, the resultant Pearson correlation coefficient COx will be near zero in case of intact CA but around 1 in case of impaired CA. Binning the data in pressure increments of e.g. 5 mmHg allows to visually determine the LLA and ULA thresholds, by identifying the step increase in COx. As alternative technique of differentiating the intact and impaired CBF autoregulation zones, the authors developed a novel model using two automated data clustering methods based on historical raw (unbinned) data from porcine experiments. For this purpose, seven pigs had been exposed to different interventions including hyper- and hypoventilation, lung recruitment manoeuvres, acute hypoxia, and haemorrhagic shock. They used a rather high COx threshold of 0.5 to differentiate intact from impaired CA in order to reduce the influence of noisy values tending to zero. Subsequently, they compared both methods of determining the LLA and found a good agreement. Both of their clustering methods revealed very distinct LLA thresholds (while ULA threshold could not be determined due to lack of data), which were comparable albeit slightly lower than those derived from the traditionally binned data algorithm. The authors conclude that their new method of determining the LLA of CA is feasible and may be considered an alternative method in continuous NIRS-based CA monitoring, particularly in noisy environments (in terms of data purity) such as those frequently encountered in clinical practice. Furthermore, their methods might also apply to other correlation-based methods of determining CA thresholds, such as the Mx or PRx modalities mentioned earlier.

## Summary

In summary, the above-mentioned studies on ScO_2_ and CA present an update in current functional cerebral monitoring. It remains to be shown if the findings related to signal processing will find their way to clinical applicability, and if the clinical findings presented here will be reproduced in larger clinical trials. Nevertheless, the JCMC has established its leading role as platform for research related to the topics of ScO_2_ and CA monitoring.
